# Adolescent Flood Exposure and Cognitive Aging: Trajectory Patterns Among Middle-Aged and Older Adults in China

**DOI:** 10.1093/geroni/igaf122.811

**Published:** 2025-12-31

**Authors:** Yunchen Ruan, Shiqi Lin, Yexin Zheng

**Affiliations:** Fuzhou University, Fuzhou, Fujian, China (People’s Republic); Fuzhou University, Fuzhou, Fujian, China (People’s Republic); Fujian Normal University, Fuzhou, Fujian, China (People’s Republic)

## Abstract

**Objective:**

This study aims to investigate the relationship between adolescent flood exposure and cognitive function trajectories among middle-aged and older adults in China from a dynamic perspective. The study is intended to address the gap in research on the long-term effects of flood exposure on cognitive function.

**Methods:**

A total of 10,352 adults aged 45 and older participating in the 2011, 2013, 2015, 2018, and 2020 waves of the China Health and Retirement Longitudinal Study were analyzed. Cognitive function trajectories were analyzed using group-based trajectory modeling. The associations between trajectory memberships and adolescent flood exposure were assessed using multinomial logistic regression.

**Results:**

Three distinct cognitive function trajectories were identified: “low start–slow decline” (18.36%), “moderate start–fast decline” (44.34%), and “high start–accelerated decline” (37.20%). After adjusting for sociodemographic variables, economic status, and health conditions, adolescent flood exposure was inversely associated with unfavorable cognitive trajectories (RRR = 0.56, 95% CI: 0.42–0.75). Mediation analysis via the Karlson–Holm–Breen method indicated that socioeconomic status significantly mediated this relationship, with years of schooling showing the strongest explanatory power (29.3%).

**Conclusions:**

Adolescent flood exposure is associated with cognitive decline trajectories in middle and old age. Disaster response strategies should prioritize long-term health benefits, enhance socioeconomic policies, and implement targeted interventions for high-risk populations. Keywords: adolescent flood exposure, cognitive aging, group-based trajectory modeling, cognitive function trajectories, Chinese middle-aged and older adults

